# Radiation Tolerance of Nanopore Sequencing Technology for Life Detection on Mars and Europa

**DOI:** 10.1038/s41598-019-41488-4

**Published:** 2019-03-29

**Authors:** Mark A. Sutton, Aaron S. Burton, Elena Zaikova, Ryan E. Sutton, William B. Brinckerhoff, Julie G. Bevilacqua, Margaret M. Weng, Michael J. Mumma, Sarah Stewart Johnson

**Affiliations:** 1 Solar System Exploration Division and Goddard Center for Astrobiology, NASA Goddard Space Flight Center, Greenbelt, MD 20771 USA; 2 Wichita State University, Wichita, KS 67260 USA; 3 Astromaterials Research and Exploration Science Division, NASA Johnson Space Center, Houston, TX 77058 USA; 4 Department of Biology, Georgetown University, Washington, DC 20057 USA; 5 Google, Boulder, CO 80301 USA; 6 Department of Earth and Planetary Science, Washington University in St. Louis, St. Louis, MO 63130 USA; 7 Science, Technology, and International Affairs Program, Georgetown University, Washington, DC 20057 USA

## Abstract

The search for life beyond Earth is a key motivator in space exploration. Informational polymers, like DNA and RNA, are key biosignatures for life as we know it. The MinION is a miniature DNA sequencer based on versatile nanopore technology that could be implemented on future planetary missions. A critical unanswered question is whether the MinION and its protein-based nanopores can withstand increased radiation exposure outside Earth’s shielding magnetic field. We evaluated the effects of ionizing radiation on the MinION platform – including flow cells, reagents, and hardware – and discovered limited performance loss when exposed to ionizing doses comparable to a mission to Mars. Targets with harsher radiation environments, like Europa, would require improved radiation resistance via additional shielding or design refinements.

## Introduction

The search for life beyond Earth is at the heart of NASA’s solar system exploration strategy. “Ocean worlds” like Europa are now within reach of current technology, and Mars continues to be a prime target for astrobiology. One approach to finding life involves detection of informational polymers like deoxyribonucleic acid (DNA) and ribonucleic acid (RNA) that are used by terrestrial organisms to encode information and to faithfully replicate it^1^. These polymers are highly definitive signatures for life as we know it, and life on Mars, if it exists, may share common ancestry with life on Earth. Alternatively, structural variants of DNA and RNA capable of storing genetic information – collectively termed xenonucleic acids (XNAs) – have been successfully synthesized in the lab^2^. Components of these nucleic acids, namely, nucleobases, sugars, and phosphorous-containing minerals are found in certain meteorites, indicating that they could be widespread in the solar system^3–6^.

Emerging nanopore technology has shown great promise for terrestrial nucleic acid sequencing^7^ but differs from traditional sequencing techniques in that it does not require amplification of DNA or RNA before analysis. This makes the technology more versatile, potentially enabling the detection and analysis of other charged polymers including XNA, proteins, and lipids^8^, though further research into this use of the technology will be required to enable such analyses. Additionally, advances in miniaturization have opened new possibilities for sequencing native nucleic acids in remote field environments^9^, and life detection instruments designed around protein-based nanopore sequencing are being developed^10^. A new life detection concept, utilizing sequencing, short oligos, and a proximity ligation assay, has also been proposed as a means to fingerprint patterns of chemical complexity, regardless of underlying biochemistry^11^.

A possible candidate instrument could be Oxford Nanopore Technologies’ (ONT) miniature nanopore-based device called the MinION^TM^ that is capable of genomic sequencing. Weighing only 85 grams, the device features nanometer-scale holes (called nanopores) in an electrically resistive membrane. In the presence of an ionic solution, an applied electrical potential causes ions to flow through the nanopores. The flow of ions generates a measurable electric current; the applied potential can also drive single-strand DNA (ssDNA) through the nanopores, thereby creating characteristic modulations of the ion current.

**Figure 1:**
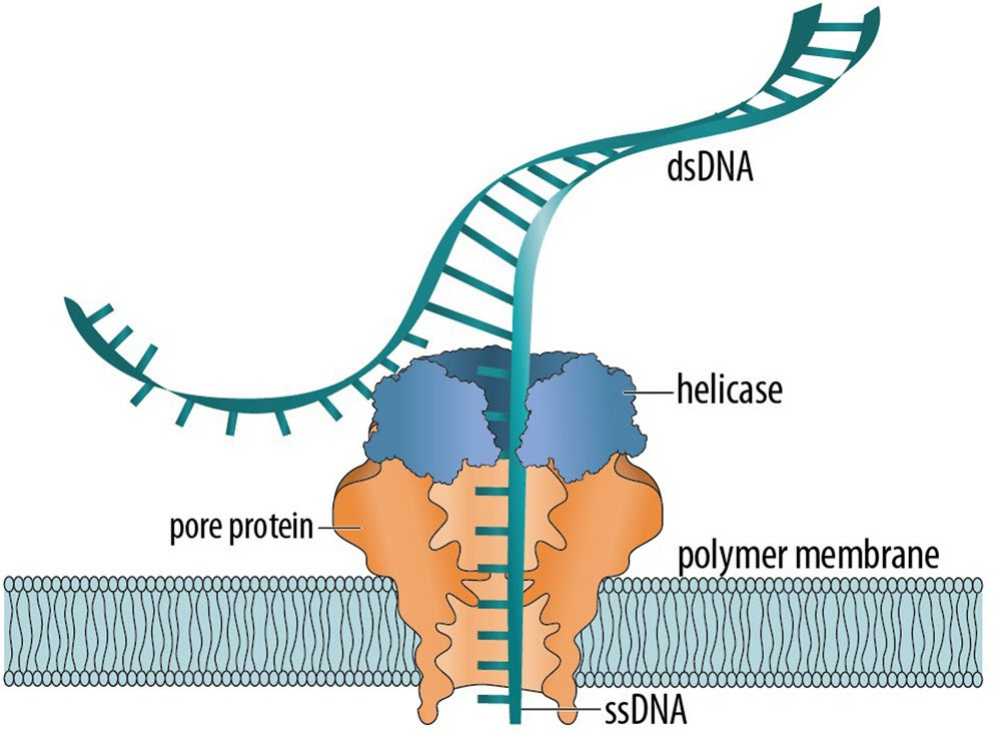
Schematic representation of a DNA molecule translocating a protein nanopore. The double-stranded DNA (dsDNA) is split by a helicase enzyme, allowing only a single strand (ssDNA) to pass while slowing it enough to achieve sufficient resolution for sequencing.

Nanopore sensors for the analysis of DNA are generated by embedding pore proteins in a synthetic polymer membrane^12^, and the MinION uses this approach. Sequencing is accomplished by pairing input double-strand DNA (dsDNA) with a helicase enzyme, which slows the polymer’s translocation through the nanopore and allows only a single strand to pass at a time (Fig. 1). Solid-state alternatives to protein-based instruments are being developed for planetary exploration^13^, but they have not yet matched their protein-based counterparts in performance.

**Figure 2:**
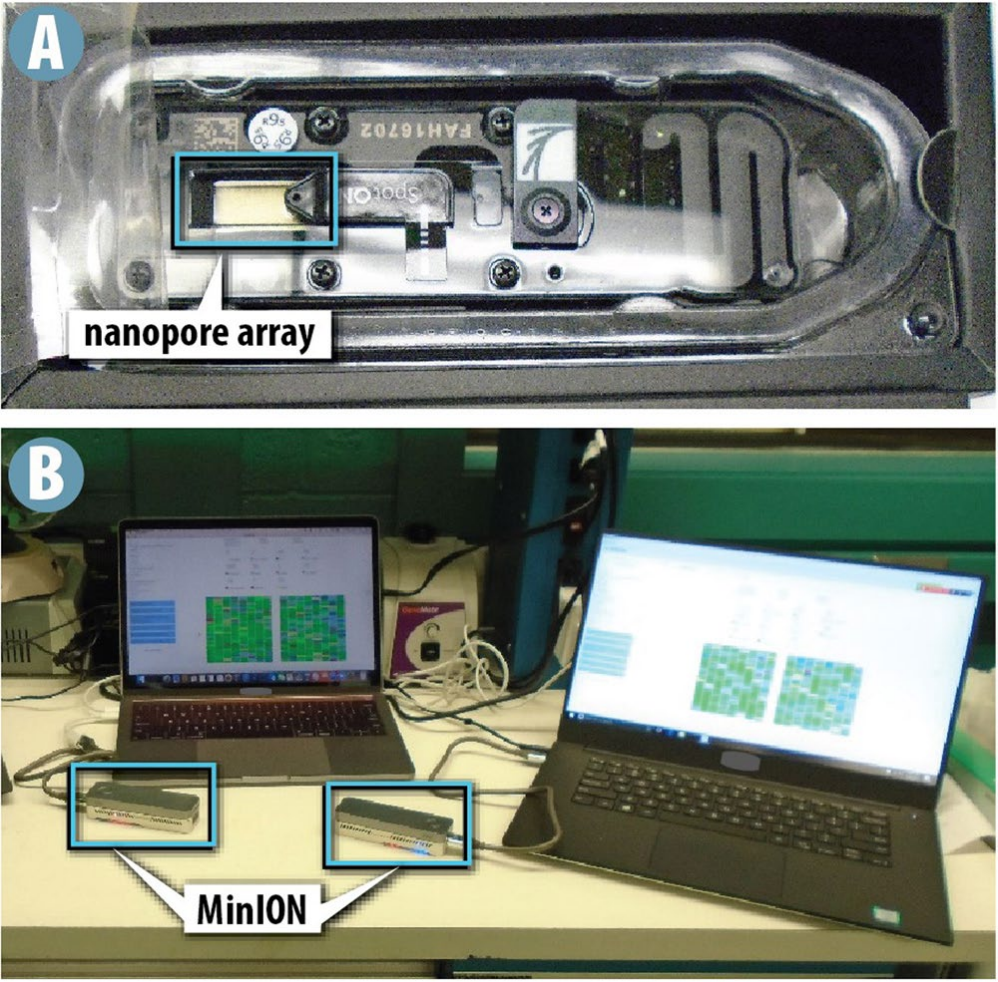
The MinION sequencer. (**a**) A typical MinION flow cell. The flow cell contains an array with sufficient space for 2048 nanopores. (**b**) Two MinION devices sequencing DNA powered via USB laptop connection.

A significant concern for the MinION’s use on a planetary mission is whether the protein and membrane-based nanopores are stable enough to withstand the temperature extremes, reduced gravity conditions, increased radiation, and vastly different atmospheric conditions that would be experienced during transit to and while on the surface of other planets upon landing. Research is being conducted to assess the effects of these conditions on nanopore sequencers. For example, a MinION was used to successfully sequence DNA onboard the International Space Station (ISS) in a micro-gravity environment^14^. Importantly, these nanopores showed no decrease in performance compared to ground controls. Here we tested the MinION platform against ionizing radiation at mission-relevant doses from a high-energy gamma ray source. Specifically, we irradiated three major components of the MinION platform: first, consumable chips known as flow cells that contain the nanopores (Fig. 2a); second, a two-reagent kit including fragmentation (FRM) and rapid adapter (RAD) reagents used to prepare DNA for sequencing; and third, the reusable MinION hardware (Fig. 2b). Each flow cell can contain up to 2048 nanopores, 512 of which may be monitored simultaneously. In the present experiments, each component was isolated and tested individually at a range of radiation doses from 10 to 3000 silicon-equivalent gray. For reference, the radiation assessment detector onboard the Mars Science Laboratory (MSL) received an average of 332 microgray per day in its silicon detector during its journey to Mars^15^, giving an approximate total dose for a typical 180-day flight of less than 0.1 gray, while instruments intended for exploration of Europa are expected to receive 1500 gray in the interior of the spacecraft over the course of the mission^16^.

**Figure 3:**
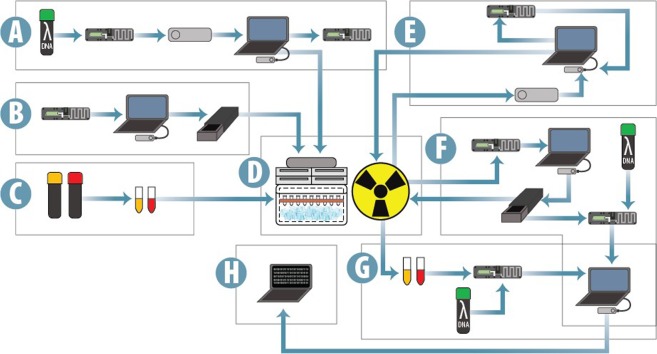
The experimental workflow. (**A**) Prior to the first irradiation, sequencing runs were attempted on both control and experimental MinION devices with lambda DNA libraries. Flow cells were removed from the MinION and stored at room temperature prior to the first irradiation. (**B**) Platform QCs were conducted on all flow cells prior to the first radiation dose. (**C**) Reagents (RAD and FRM) were aliquoted in 5 microliter volumes into 0.2 milliliter thin-walled PCR tubes and placed in the polystyrene cooler over dry ice. A total of 12 RAD aliquots (9 experimental and 3 control) and two FRM aliquots (1 experimental and 1 control) were made. (**D**) MinION hardware, flow cells, and reagents were irradiated simultaneously by a high energy gamma ray source. (**E**) The experimental MinION was removed from the chamber and short sequencing runs attempted on it and the control at cumulative doses of 250 (from the previous irradiation), 400, 600, 750, 1500, and 3000 gray. Flow cells were removed from both devices for irradiations. (**F**) One flow cell was dedicated to platform QCs at doses of 150, 300, 400, 500, 600, and 750 gray. This flow cell was placed back in the chamber after each QC. Additionally, platform QCs were conducted on three flow cells following doses of 50, 300, and 500 gray. Sequencing of prepared lambda DNA was then attempted on all flow cells. (**G**) One tube of RAD reagent was removed from the chamber and stored over dry ice in a control foam container at doses of 10, 50, 100, 150, 300, 400, 750, 1500, and 3000 gray. The tube of FRM was removed from the chamber at 400 gray. All RAD reagents were then barcoded and sequenced on a single flow cell. The two FRM reagents were sequenced on separate flow cells. (**H**) Raw sequencing data was basecalled by Oxford Nanopore’s Albacore software, aligned to the lambda genome with NanoOK, and further analyzed by custom Python code.

Performance of all components was assessed via sequencing of lambda phage virus genomic DNA (Fig. 3). Flow cells were further evaluated using a self-diagnostic protocol known as a platform quality control (QC) whereby a potential is applied across the nanopore membrane and the resulting ionic current used to assess and report an estimated number of viable nanopores remaining. Sequencing data were analyzed for performance metrics including the quality of the alignments to the lambda genome as well as “skips” and “stays” that indicate the quality of the DNA reads produced as previously described by McIntyre *et al*.^17^. We note that future nanopore-based technologies will likely still use many of the same critical design elements as the MinION and identify components that would need to be redesigned for successful operation in space.

## Results

### Irradiation effects on flow cells

To test the effects of irradiation on flow cell and MinION function, four model R9.5 flow cells were irradiated, and two additional flow cells were used for treatment controls. One was dedicated to repeated platform QC experiments at doses of 150, 300, 400, 500, 600, and 750 gray while the remaining three were removed from the radiation chamber at 50, 300, and 500 gray for sequencing. One control was subjected to repeated platform QCs alongside the dedicated QC flow cell.

Platform QC data for all flow cells are listed in Tables 1 and 2. Typical for R9.5 flow cells, all flow cells used in this study had between 977 and 1246 active pores prior to the first irradiation. Some variability in pore responses to radiation was observed, as the dedicated QC flow cell reported only 282 active pores after a 300-gray dose while the flow cell removed for sequencing at 300 gray reported 1118 active pores (Table 2). Importantly, no drop in active nanopores was observed at 50 gray and only a modest loss observed at 150 gray. The flow cell removed for sequencing at 500 gray could no longer be recognized by MinKNOW software and a platform QC could not be conducted. A QC was not conducted after the final irradiation on the control flow cell.

**Table 1:** Platform QC Flow Cells.

Flow Cell	Active Nanopores Reported								
	Initial	150 gray	300 gray	400 gray	500 gray	600 gray	750 gray	Pre-sequencing QC	Post-sequencing QC
Control	1240	1236	1227	1213	1226	1213	1220	1218	660
Irradiated	1246	1081	282	139	348	221	19	52	206

**Table 2:** Sequencing Flow Cells.

Flow Cell Dose (gray)	Active Nanopores Reported			
	Pre-irradiation QC	Post-irradiation QC	Pre-sequencing QC	Post-sequencing QC
0 (Control)	1051	N/A	1042	334
50	977	979	983	232
300	1116	1118	1116	220
500	1161	N/A	102	345

**Figure 4:**
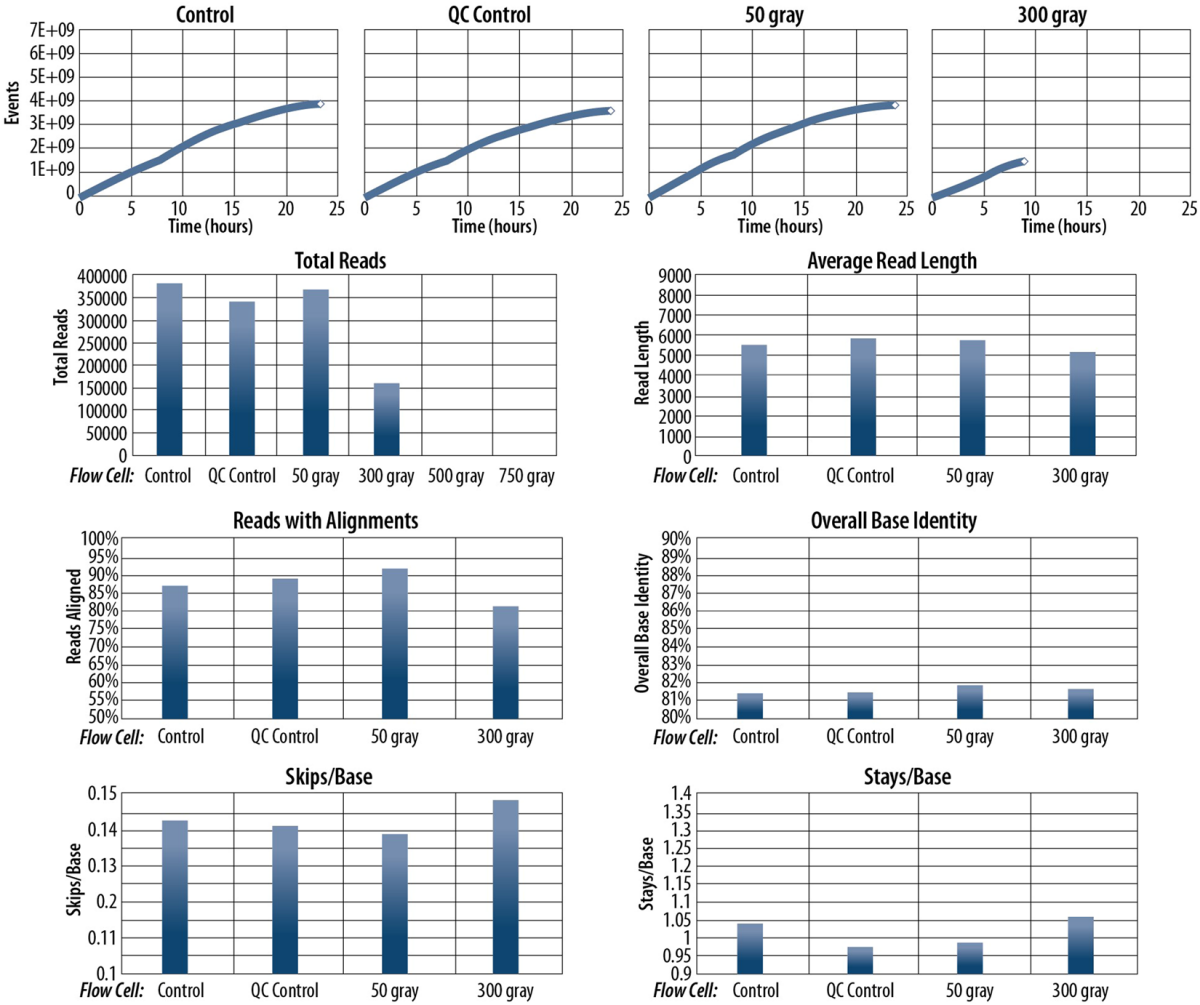
Sequencing alignment and quality analyses for flow cell experiments. No data is presented for flow cells at 500 or 750 gray as neither produced any reads during sequencing.

To determine whether the active pores remaining after irradiation function as expected, we sequenced a lambda phage genome library on the irradiated and corresponding control flow cells. Sequencing was successful on both 50-gray and 300-gray flow cells (Fig. 4 and Supplementary Table S1). At 50 gray, no decrease in sequencing throughput or quality was observed. Over 24 hours, the 50-gray flow cell produced 364806 reads with 91.66% aligning with the lambda genome compared with 376358 at 86.92% and 342667 at 88.93% for both controls. The 300-gray flow cell sequenced for approximately 8 hours before it abruptly stopped producing reads. The exact cause of this is not known, but it does appear to correspond with the multiplexing unit (MUX) change that occurs at 8-hour intervals. (During a MUX change, the MinKNOW software begins to monitor a new set of up to 512 nanopores). Fewer reads aligned to the lambda genome (81.09%) than either control or 50-gray flow cells and skips and stays were both slightly elevated. The average read length was significantly shorter than the other three flow cells, but this may be partially explained by the lack of reads obtained after 8 hours as shorter DNA fragments are preferentially sequenced early in a run. Interestingly, the quality of the alignments does not appear to have been significantly affected, as indicated by a slightly higher than control base identity (81.63%). The 500-gray flow cell was recognized by MinKNOW for a pre-sequencing QC but reported only 102 active nanopores compared to 1161 before irradiation. Additionally, both it and the flow cell used exclusively for platform QCs irradiated to 750 gray failed to produce any reads over the 24-hour runs. Notably, all flow cells producing reads yielded sequencing data that completely covered the lambda genome. Genome coverage maps are available in Supplementary Fig. S1.

### Irradiation effects on reagents

A total of nine 5-microliter aliquots of RAD reagent and one of FRM reagent were irradiated over dry ice in thin-walled PCR tubes, with three aliquots of RAD and one of FRM kept aside for controls. Tubes of RAD were removed from the radiation chamber at 10, 50, 100, 150, 300, 400, 750, 1500, and 3000 gray with the FRM removed at 400 gray. All reagents were used to prepare lambda DNA libraries. RAD reagents were sequenced on a single flow cell using uniquely barcoded libraries while the FRM was sequenced on a separate flow cell.

**Figure 5:**
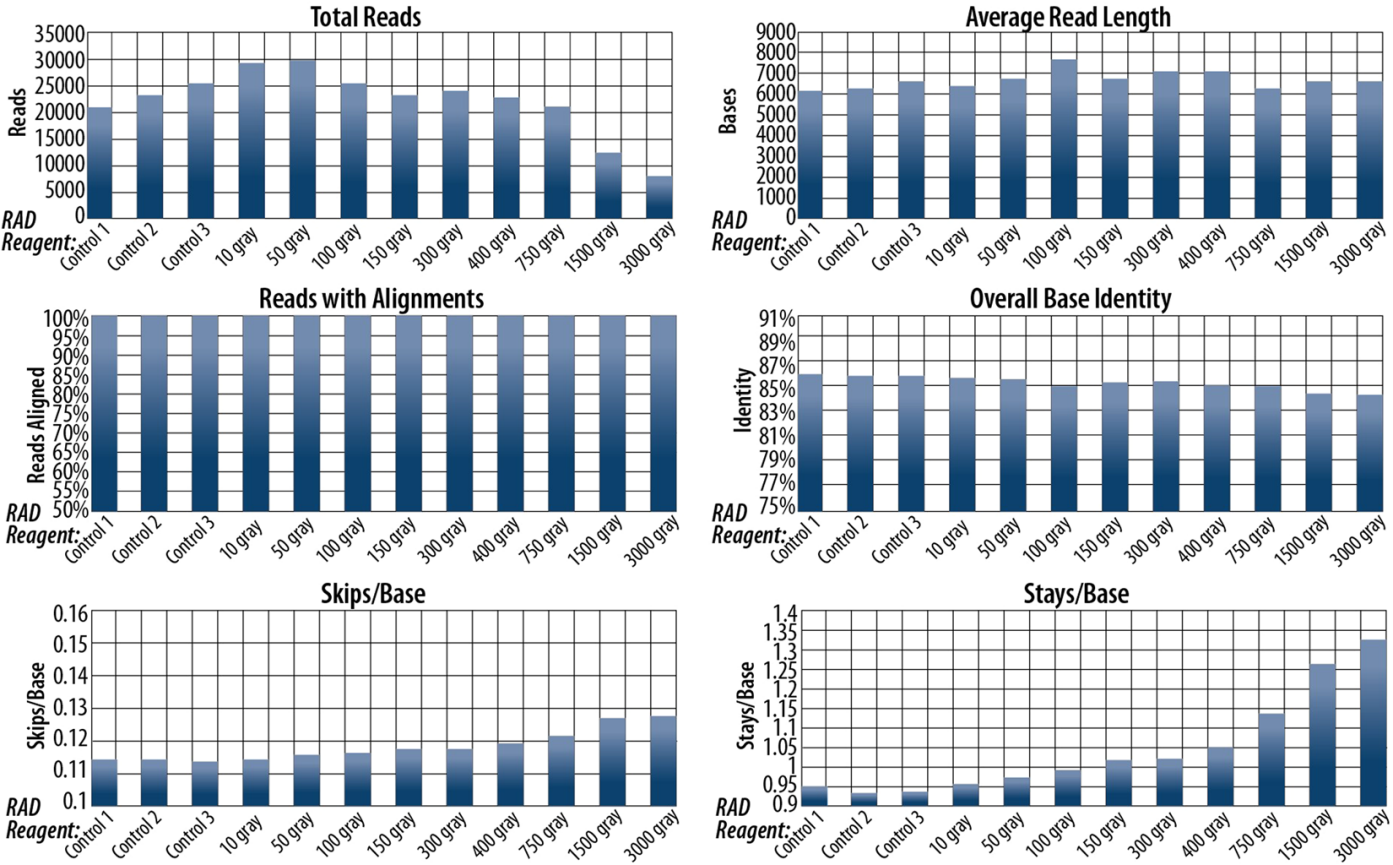
Sequencing alignment and quality analyses for RAD reagent experiments. Note that reads produced by all reagents showed nearly 100% of reads having alignments to the lambda genome. This was expected due to lower quality reads not being identified with a barcode by the basecalling software.

**Figure 6:**
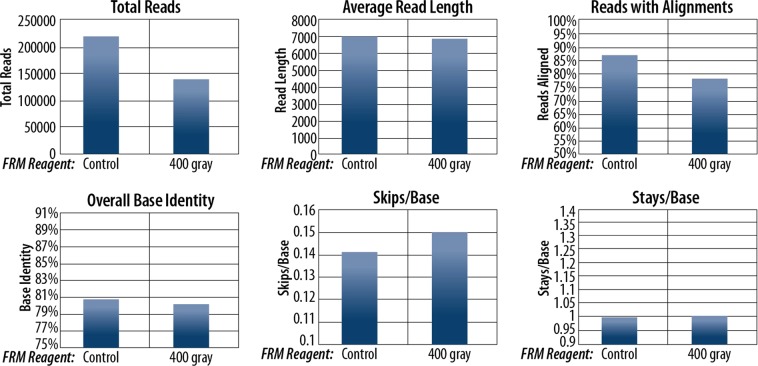
Sequencing alignment and quality analyses for FRM reagent experiments.

Analyses for RAD reagent sequencing are presented in Fig. 5 (tabular format provided in Supplementary Table S2). There was no drop in performance below that of the worst performing control for reagents up to and including 750 gray. A wide range of read productions was observed, even between controls. This may be due to deviations from the nominally equal concentrations of each reagent in the final sequencing mixture. Alignment data were similar across all doses, as expected due to low quality reads not being classified by basecalling software. Even so, a steady decrease in performance was observed with increasing dose as indicated by elevated skips and stays as well as decreasing base identity. Average read lengths were similar across all doses. All RAD reagents produced complete coverage of the lambda genome. Results of FRM reagent sequencing are shown in Fig. 6 (tabular format provided in Supplementary Table S3). The reagent at 400 gray showed decreased performance across all reported metrics. Skips and stays were elevated while the percentage of reads with alignments, base identity, and total number of reads were all lower than the control. However, both still fully covered the lambda genome (Supplementary Figs S2 and S3).

The time dependence of skips, stays, read length, and the electrical current level detected while sequencing for both flow cell and reagent experiments were also produced by our analysis and are shown in Supplementary Figs S4–S6.

### Irradiation effects on *MinION* hardware

One MinION Mk1B irradiated previously to 250 gray was further irradiated to cumulative doses of 400, 600, 750, 1500, and 3000 gray. Between each dose, brief sequencing runs of approximately 15 minutes were attempted on it and a control device using ligation-prepared lambda DNA. Both control and experimental MinIONs produced few reads (less than 250) for each run. Analysis of the data revealed poor alignment to the lambda genome and generally low read quality. This was most likely the result of an unsuccessful preparation of the input DNA, not the failure of MinION hardware. There was insufficient time during the irradiation experiments to prepare secondary lambda DNA; thus, the experiment continued as scheduled without replacing the failed DNA library. There were no noticeable changes in performance between the devices up to and including a dose of 750 gray. Following the 1500-gray dose, the irradiated device continued to sequence normally but had to be reconnected to its laptop multiple times before it was recognized by the MinKNOW software. After the 3000-gray dose, the device could not be recognized. Analyses for these sequencing runs are presented in Supplementary Table S4.

## Discussion

Of the three major MinION components, flow cells exhibited the greatest decline in function due to irradiation. Further studies are needed to determine whether the observed decline was due to solid state or protein-based component failure. A new flow cell design set to be released soon called the “Flongle” may aid in these studies by allowing separate irradiation of much of the flow cell’s circuitry and protein-based components.

Both RAD and FRM reagents produced enough reads of sufficient quality to cover the lambda genome at all doses tested and RAD reagents showed only a slight decrease in performance for lower doses. Between the two reagents, FRM appears to be more susceptible to ionizing radiation, though only one aliquot of FRM was irradiated. This experiment will need to be repeated to permit more rigorous analysis of its radiation response. The MinION device proved most resilient to ionizing radiation, but further hardware tests should include separation of critical electrical components from their metallic casing, as future nanopore instruments may not share the MinION’s external design and assessment of the exposed critical electrical components is of interest.

The overall MinION platform has proven to be surprisingly robust. Considering the lightweight and compact design with minimal power requirements, it may be possible to integrate the MinION into a spaceflight instrument. Our results indicate that the MinION could be suitable for missions to Mars or other relatively nearby targets with limited modification, as all components proved to be robust at radiation doses in vast excess of those expected on a typical Mars mission, and other protein-based nanopore designs may exhibit similar radiation tolerance. In addition, The Biomolecule Sequencer project^14^ demonstrated the viability of flow cells in the space environment 6 months after production, with no apparent decrease in flow cell pore counts or sequencing performance. The transit times for the two most recent landed Mars missions, InSight and Curiosity, were approximately 6 and 9 months, respectively, suggesting that current flow cell lifetimes could be used at least during the early phases of a Mars mission without any stability improvements.

Significant loss of performance across all MinION components at radiation doses consistent with those expected on a mission to Europa suggests that significant design refinement would be required to reach targets with harsher radiation environments. Additional shielding could be an option but would come at the cost of increased volume and weight. It is also possible that the development of solid-state alternatives to protein nanopores could enhance radiation tolerance, but it is important to note that protein damage has not been isolated as the mechanism for flow cell failure.

Gamma rays have been used in this study as a first approximation of the space radiation environment. In doing so, we have limited the scope of our results to total ionizing dose effects. More in-depth studies should consider other forms of radiation such as protons, neutrons, and heavy ions. Dose rate effects should also be considered in the future.

Future studies should also consider temperature effects, both as it applies to radiation tolerance and storage conditions in transit to Mars or Europa. Flow cells are ideally stored between 4 °C and 8 °C while reagents are stored at −20 °C, as per ONT recommendations. Maintaining a suitable flow cell temperature during flight may prove difficult and exposure to freezing temperatures typically destroys the flow cell’s nanopore array, rendering it useless. Thus, alternative storage schemes should be investigated such as vitrification and the on-site assembly of the nanopore array.

Finally, preparing extraterrestrial samples for sequencing will be challenging and should be the subject of future work. Some progress has been made on developing DNA extraction procedures for Mars-like soil samples^18^, but research remains in the early stages. Extraction from liquid samples, like those expected from Europa or other icy worlds is in principle also possible, though the efficient extraction of genetic material from potentially very low biomass samples will be difficult in either case.

## Conclusion

We evaluated the performance of MinION technology as a function of radiation dosage over ranges appropriate for missions to Mars and to Europa. Flow cells produced DNA reads of sufficient quality and quantity to cover the lambda genome at doses of up to 300 gray in these initial experiments. Similarly, RAD reagents and the FRM reagent produced DNA reads of sufficient quality and quantity to cover the lambda genome at doses up to 3000 gray and 400 gray, respectively. The MinION hardware performed as expected up to and including a 750-gray dose. We conclude that the MinION instrument is robust to ionizing radiation doses associated with expeditions to Mars with limited performance loss, but targets with harsher radiation environments (like Europa) will require improved radiation resistance via additional shielding or design refinements. These results warrant further research into protein-based technologies for spaceflight instruments.

## Materials and Methods

### Irradiations

**Figure 7:**
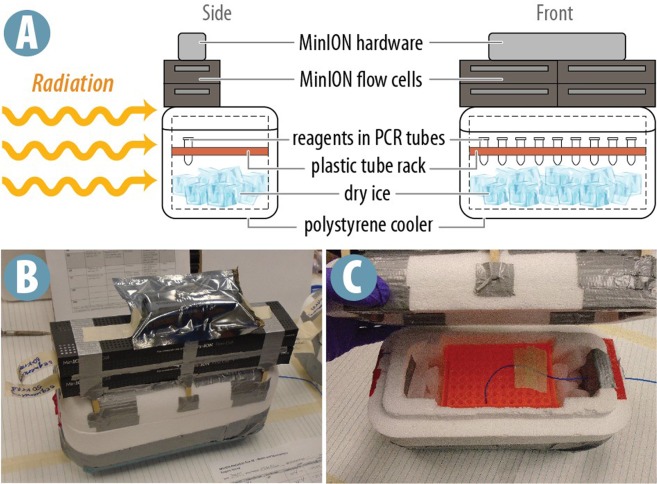
The radiation apparatus. (**A**) Schematic representation of the radiation apparatus. Reagents in PCR tubes were suspended over dry ice in a plastic tube rack enclosed by a polystyrene cooler. Flow cells in antistatic bags were placed in manufacturer boxes directly on top of the cooler. MinION hardware in an antistatic bag was placed on top of the flow cell boxes. (**B**) The radiation apparatus just prior to placement in the chamber. (**C**) The cooler opened to show the tube rack and dry ice.

Irradiations were carried out on July 30^th^, 2017 with the assistance of NASA Goddard Space Flight Center (GSFC) personnel. The high energy gamma rays used had energies around 1.25 MeV. Dosimetry was performed using 0.03 cc open air ionization probes and medical therapy dosimeters, in measurements of exposure, units of roentgen (R). Equivalence to absorbed dose (gray) depends on the photon energy spectrum and absorbing material. Silicon is a common material of interest and is one of the target materials in this test. For these irradiations an equivalence of 0.008656 gray(Si)/R was used. Dose rates were calibrated by adjusting horizontal distance from the source. Substitution dosimetry, where the dose rate at a location is measured without the target material present and then the material is put into that location, determined the appropriate distance from the radioactive source. Accumulated doses were controlled by irradiation time. All materials used to mount or enclose MinION components were chosen such that they provided only negligible shielding. Flow cells, reagents, and MinION hardware were irradiated simultaneously as shown in Fig. 7.

Reagents were aliquoted in 5-microliter volumes into 0.2-milliliter thin-walled PCR tubes and suspended over dry ice in a polystyrene cooler. Temperature in the container remained between −20 °C and −45 °C for the duration of the experiment and reagents are not expected to have thawed at any point. Two reagents from ONT’s Rapid Sequencing Kit (RAD-002) were irradiated. One tube of fragmentation mix (FRM) and 9 tubes of rapid adapter (RAD) were placed in the chamber with one tube of FRM and three tubes of RAD kept outside the chamber under similar conditions for control. The Rapid Sequencing Kit was selected for its simplified 10-minute procedure that is best suited for field applications. The FRM reagent was removed from the chamber after receiving a 400-gray dose while RAD reagents were removed sequentially at 10, 50, 100, 150, 300, 400, 750, 1500, and 3000-gray doses. Reagents removed from the chamber were quickly transferred to the control container with care taken to minimize the time both containers spent open to the ambient environment (less than 10 seconds for each transfer). Following the final irradiation, all reagents were stored at −20 °C until use.

Flow cells were sealed in antistatic bags and irradiated atop the polystyrene cooler in manufacturer packaging. Flow cells were oriented with their largest surfaces perpendicular to incoming radiation and with their nanopore arrays centrally directed. Two stacks of two flow cells were formed with each stack equidistant from the middle plane. Flow cells were model R9.5 (FLO-MIN107). Two flow cells were kept under similar conditions outside the chamber as controls. No attempt was made to control flow cell temperature during irradiation though the chamber is not expected to have varied significantly from ambient lab conditions (22–25 °C). As with the reagents, flow cells were removed from the chamber sequentially at doses of 50, 300, and 500 gray. Platform QCs were conducted on each before irradiation and after their removal. Additionally, a fourth flow cell was removed from the chamber and replaced at doses of 150, 300, 400, 500, 600, and 750 gray so that platform QC experiments could be performed after each. One of the control flow cells was subject in parallel to the same experiments. MinKNOW software version 1.7.10 was used for all platform QC experiments.

A single MinION Mk1B device was irradiated atop the stacked flow cells. It was positioned with its underside oriented toward the beam and lid closed. This position was selected because it is assumed to be the device’s most vulnerable position due to the relative lack of shielding provided by its casing. Doses were calculated from the position of the internal microchip visible through the side air vents without considering shielding from the casing. That is, the ionizing doses received by the primary electronics is expected to be less than that calculated for the flow cells and reagents. A second device was kept as a control under similar conditions at all times except for a single, short (less than 8 hours) sequencing run conducted on the control device under climate-controlled indoor conditions. Additionally, the experimental device started the experiment at 250 gray from a previous irradiation. Total doses of 250 (from the previous irradiation), 400, 600, 750, 1500, and 3000 gray were achieved. Short sequencing runs (approximately 15 minutes) were attempted simultaneously on both devices on identical 1D ligation prepared lambda phage virus genomic DNA with unused R9.5 flow cells initially and between each dose. Flow cells with loaded DNA libraries were disconnected from the MinION device during irradiation and stored under ambient conditions between doses. Sequencing was attempted using MinKNOW 1.7.10.

Dose rates were 7.1 gray per minute up to and including the 750-gray dose and 15.1 gray per minute for all remaining doses. Dose rate effects are not considered in these experiments.

### Sequencing

Sequencing was attempted on all four irradiated flow cells and both control flow cells in groups of three starting approximately one day after irradiation. Sequencing was delayed due to the failure of the preparation of the DNA libraries intended for testing the MinION hardware. DNA prepared at the same time was originally intended for sequencing on the irradiated flow cells, but new libraries were prepared to ensure sequencing success. The first group consisted of one of the control flow cells and two irradiated flow cells at 50 and 300 gray. Genomic lambda virus DNA (200 nanograms) was prepared just prior to sequencing according to ONT Rapid Sequencing Kit (RAD002) protocols with barcode 12 (BC12) fragmentation mix from the Rapid Barcoding Kit (RBK001) substituted for the normal, non-barcoded FRM reagent. Before final loading into the flow cells, all three DNA libraries were diluted with nuclease-free water to 150 microliters, combined to ensure consistency, and injected through the priming port. Sequencing was initiated simultaneously and allowed to continue for 24 hours. Runs were stopped manually and sequencing on the remaining flow cells (the control and experimental flow cells used for platform QCs as well as the 500-gray flow cell) was initiated in a similar fashion. Laptops used for sequencing included a Dell Precision 5510 (Intel CORE i7, 1TB solid-state drive, and 16GB memory), Acer Aspire ES-573 series (Intel CORE i5, 500GB solid-state drive, 8GB memory), and a MacBook Pro (Intel CORE i7, 500GB solid-state drive, 16GB memory) all running MinKNOW 1.7.10. All MinION devices were model Mk1B.

Each of the 12 RAD reagents (nine irradiated, three control) were used to prepare barcoded lambda DNA libraries using the ONT Rapid Barcoding Kit (RBK-001) and following standard protocols with the exception that each of the 12 libraries were fully prepared before pooling in a total of 10 microliters. The Rapid Barcoding Kit shares all critical reagents and preparation steps with the Rapid Sequencing Kit but includes 12 fragmentation mixes, each tagged with a unique DNA sequence that can be used to identify the library during post-sequencing analysis. Each RAD reagent was given a unique barcode. The pooled prepared DNA was loaded via the SpotON port into an unused flow cell and allowed to sequence for 24 hours. Control and irradiated FRM reagents were then used to prepare lambda DNA via ONT Rapid Sequencing Kit protocols except that both libraries were diluted to 150 microliters with nuclease-free water and loaded through the priming port. Sequencing was initiated simultaneously and allowed to continue for 24 hours. Sequencing of RAD reagents began six days following irradiation.

### Data analysis

All raw sequencing data were basecalled using ONT Albacore software (version 1.2.6), and alignments were performed using NanoOK software (version 1.25) selecting the “LAST” aligner option^19^. The reference genome for all alignments was NC_001416.1. Relevant analyses produced by NanoOK include the total number of reads produced, the percentage of reads aligning to the reference genome, overall base identity, and reference genome coverage. The total number of reads corresponds to the number of DNA strands passing through the nanopore. Base identity is the percentage of bases analyzed that align with the reference genome, and reference genome coverage is a measure of how many times different sections of the reference genome were aligned to by the analyzed DNA. Complete coverage indicates that the entire reference genome was aligned to at least once.

Custom-developed Python code was used to further analyze basecalled data to obtain events, read length, skips per base, and stays per base analyses. The MinION detects five bases of a DNA strand at a time, and a single event corresponds to the detection of one set of five bases. Read length is the number of bases detected for a single strand of DNA passing through the nanopore. Skips and stays are indicators of read quality. Skips occur when expected bases in the DNA sequence do not correspond to an event while stays occur when multiple events correspond to the same set of bases.

### Code Availability

Python code used for analysis of basecalled.fast5 files is available at https://github.com/sutton304803/fast5-analysis-code. Opensource code upon which our code is based is available at https://github.com/PianoRoKR/time-chunked-hdf5-analysis-framework.

## Supplementary information

Supplementary Information

## Data Availability

All data generated and analyzed during this study, including raw.fast5 files, are available from the corresponding author upon reasonable request. Full NanoOK report documents are additionally available at the following address: https://figshare.com/articles/Radiation_Tolerance_of_Nanopore *_Sequencing_Technology/7575575*. All other analyzed data are included in this published article and its supporting supplementary information file.
