# Supplementary material for: Radiation Tolerance of Nanopore Sequencing Technology for Life Detection on Mars and Europa
Source: Sci Rep. 2019 Mar 29;9:5370. doi: 10.1038/s41598-019-41488-4 (PMC6441015; doi:10.1038/s41598-019-41488-4)
Supplement: Supplementary file 1 — Supplementary Information [file 41598_2019_41488_MOESM1_ESM.pdf]

Radiation Tolerance of Nanopore Sequencing Technology for Life Detection on Mars and Europa

Mark A. Sutton[1,2], Aaron S. Burton[3], Elena Zaikova[4], Ryan E. Sutton[5], William B. Brinckerhoff[1], Julie G. Bevilacqua[4], Margaret M. Weng[6], Michael J. Mumma[1], Sarah Stewart Johnson[4,7]*

[1]Solar System Exploration Division and Goddard Center for Astrobiology, NASA Goddard Space Flight Center, Greenbelt, MD 20771; [2]Wichita State University, Wichita, KS 67260; [3]Astromaterials Research and Exploration Science Division, NASA Johnson Space Center, Houston, TX 77058; [4]Department of Biology, Georgetown University, Washington, D.C. 20057; [5]Google, Boulder, CO 80301; [6]Department of Earth and Planetary Science, Washington University in St. Louis, St. Louis, MO 63130; [7]Science, Technology, and International Affairs Program, Georgetown University, Washington, D.C. 20057

*to whom correspondence should be addressed: sarah.johnson@georgetown.edu

Supplementary Information

Supplementary Table S1

Flow Cell	Reads Produced	Reads with Alignments	Overall Base Identity	Skips per Base	Stays per Base	Average Read Length
Control	376358	86.92%	81.40%	0.142701	1.038575	5512.41
QC Control	342667	88.93%	81.45%	0.141041	0.973341	5793.68
50 gray	364806	91.66%	81.83%	0.138916	0.986213	5717.15
300 gray	159217	81.09%	81.63%	0.148277	1.056920	5166.19
500 gray	No Data					
750 gray	No Data					

Supplementary Table S2

RAD Reagent	Reads Produced	Reads with Alignments	Overall Base Identity	Skips per Base	Stays per Base	Average Read Length
Control 1	20854	99.75%	85.88%	0.114519	0.951390	6135.85
Control 2	23316	99.74%	85.76%	0.114305	0.933882	6284.11
Control 3	25488	99.71%	85.76%	0.113953	0.937566	6543.03
10 gray	29588	99.69%	85.62%	0.114492	0.954083	6387.99
50 gray	30040	99.66%	85.51%	0.115776	0.973833	6748.33
100 gray	25420	99.70%	85.00%	0.116176	0.993173	7650.96
150 gray	23432	99.69%	85.27%	0.117498	1.018458	6798.66
300 gray	24186	99.70%	85.32%	0.117394	1.020409	7116.02
400 gray	22930	99.65%	85.02%	0.119586	1.050160	7090.43
750 gray	21286	99.61%	84.98%	0.121715	1.136221	6403.95
1500 gray	12586	99.67%	84.40%	0.126965	1.262787	6670.16
3000 gray	8090	99.70%	84.28%	0.127719	1.325033	6632.01

Supplementary Table S3

FRM Reagent	Reads Produced	Reads with Alignments	Overall Base Identity	Skips per Base	Stays per Base	Average Read Length
Control	217867	86.98%	80.76%	0.141103	0.997552	7103.31
400 gray	142032	77.85%	80.12%	0.150688	1.003970	6877.97

Note: skips and stays are truncated at 6 decimal places

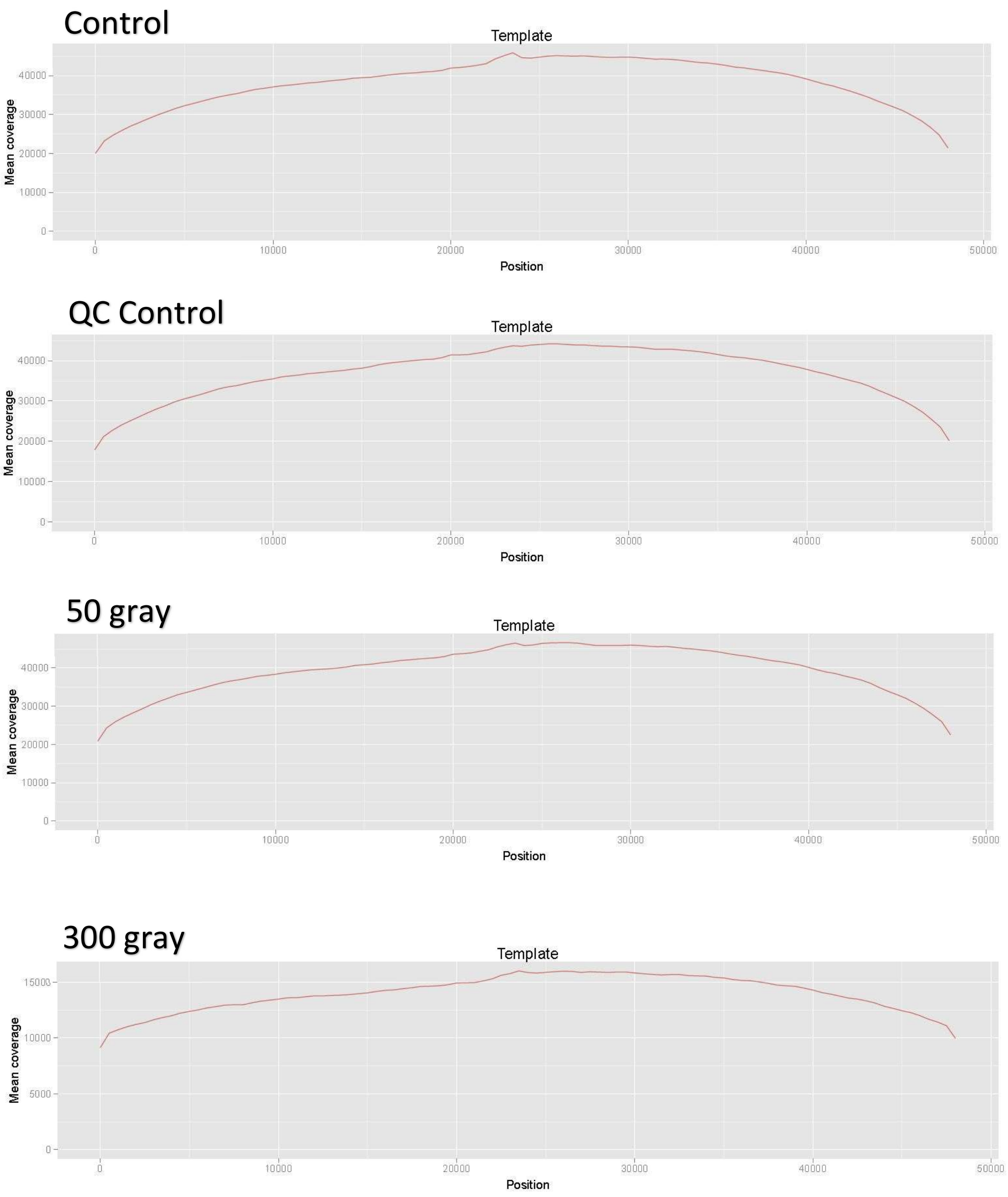

Supplementary Figure S1. Lambda genome coverage maps for flow cell experiments. The lambda genome is approximately 48,000 base pairs long.

Barcode 12 - Control 1

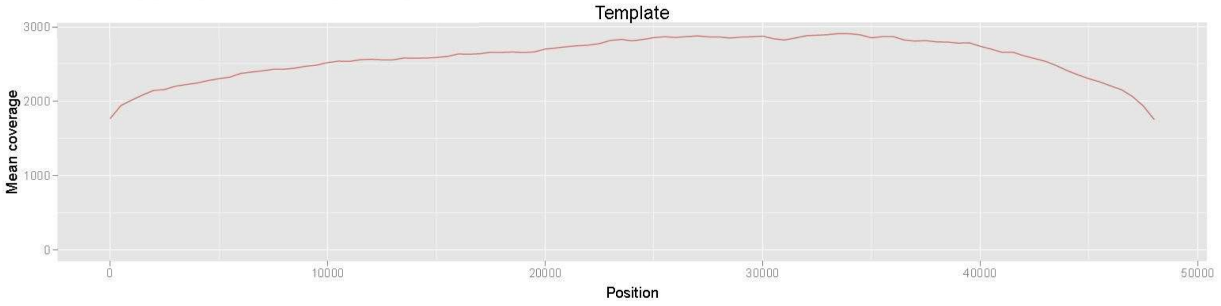

Barcode 11 - Control 2

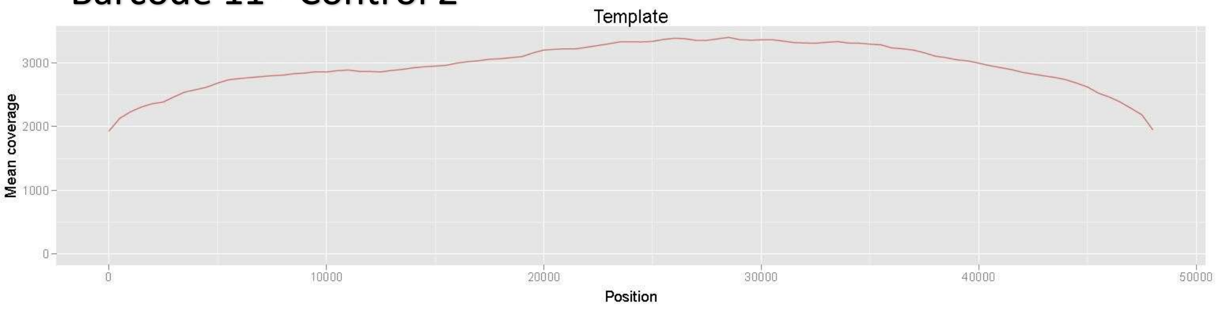

Barcode 10 - Control 3

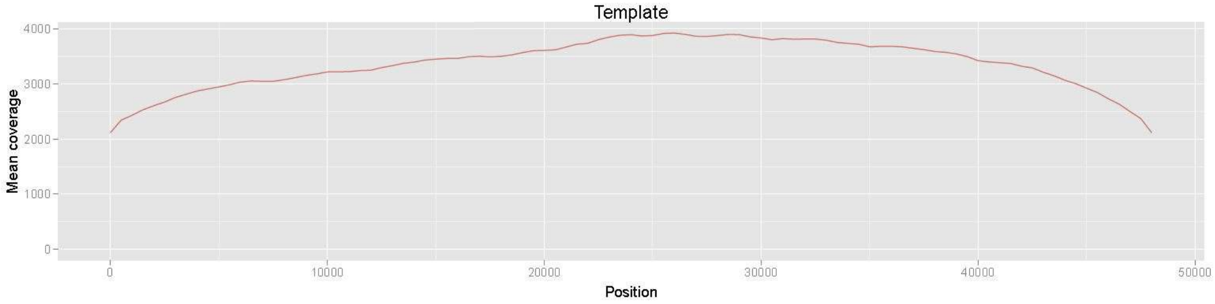

Supplementary Figure S2. Lambda genome coverage maps for RAD reagent experiments.

Barcode 1 – 10 gray

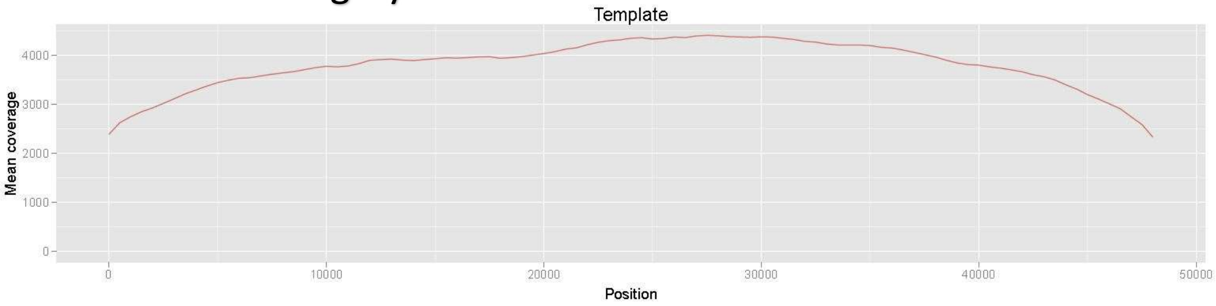

Barcode 2 – 50 gray

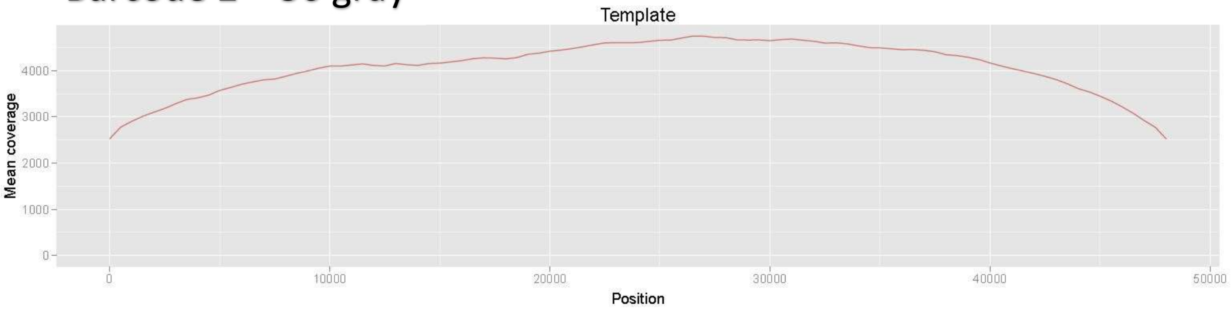

Barcode 3 – 100 gray

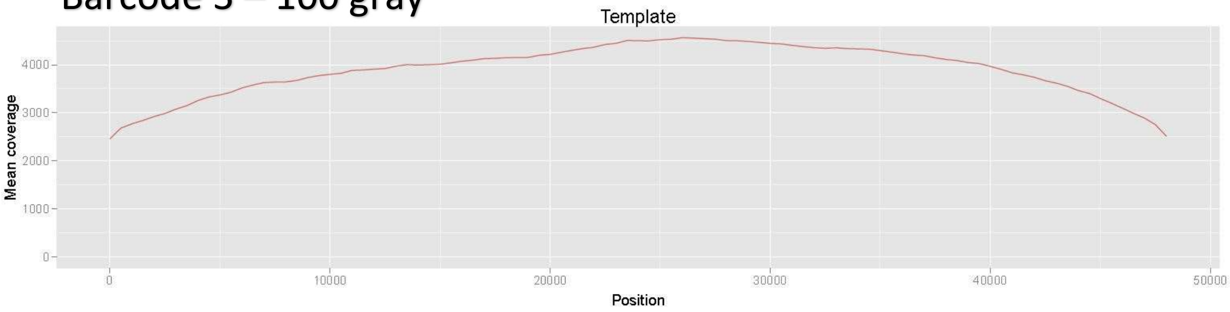

Supplementary Figure S2 (continued)

Barcode 4 – 150 gray

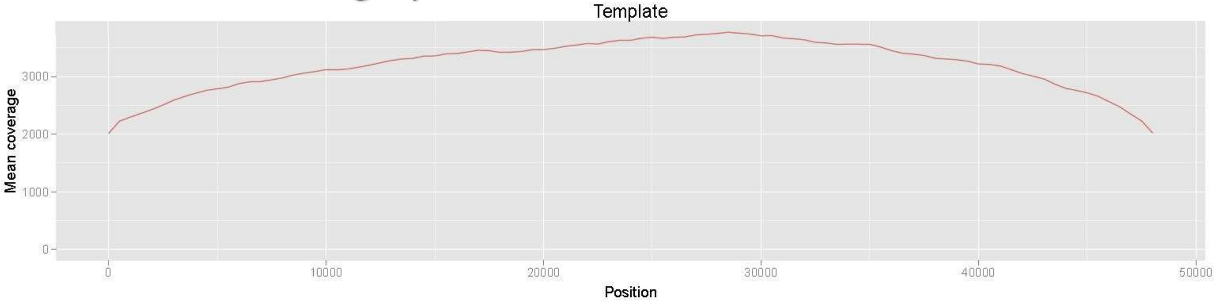

Barcode 5 – 300 gray

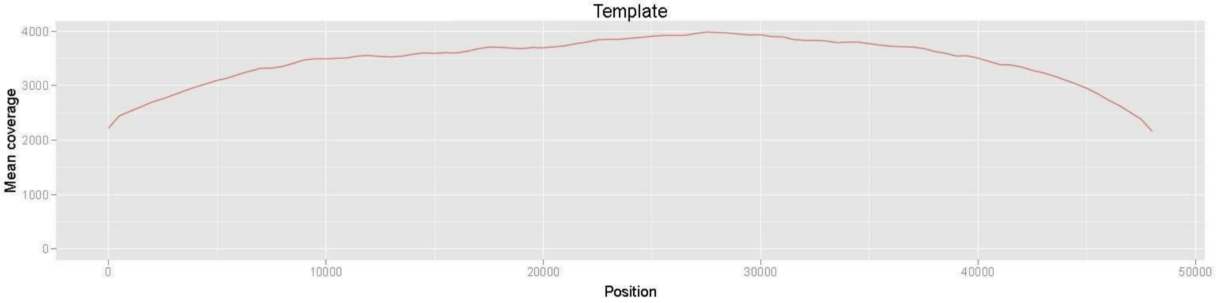

Barcode 6 – 400 gray

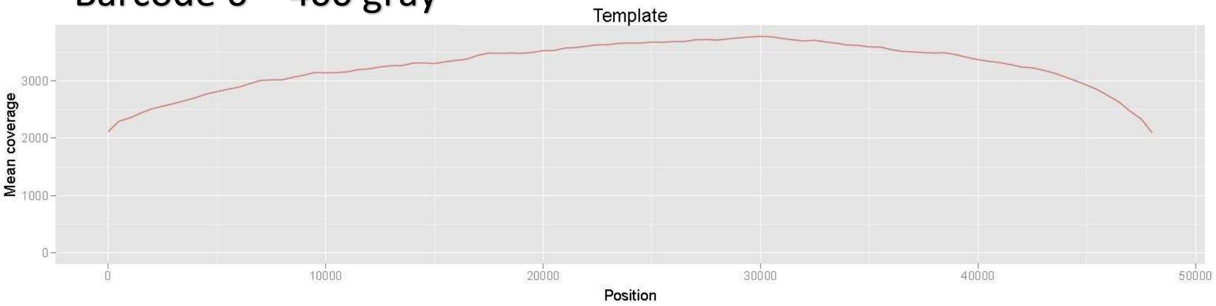

Supplementary Figure S2 (continued)

Barcode 7 – 750 gray

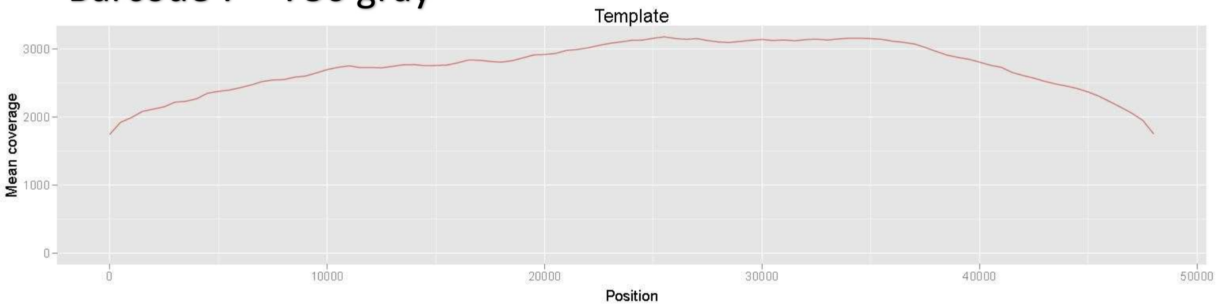

Barcode 8 – 1500 gray

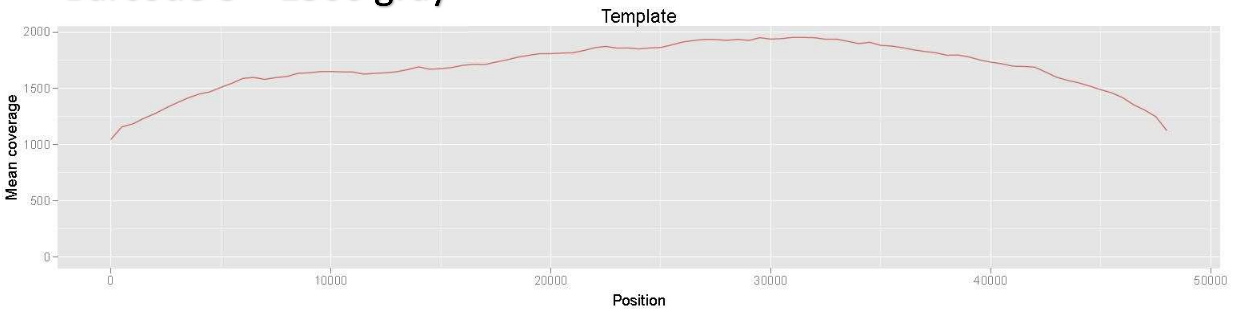

Barcode 9 – 3000 gray

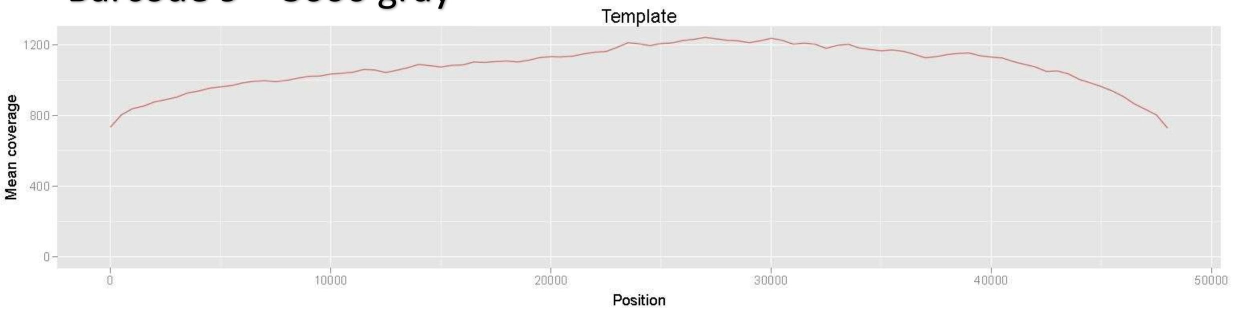

Supplementary Figure S2 (continued)

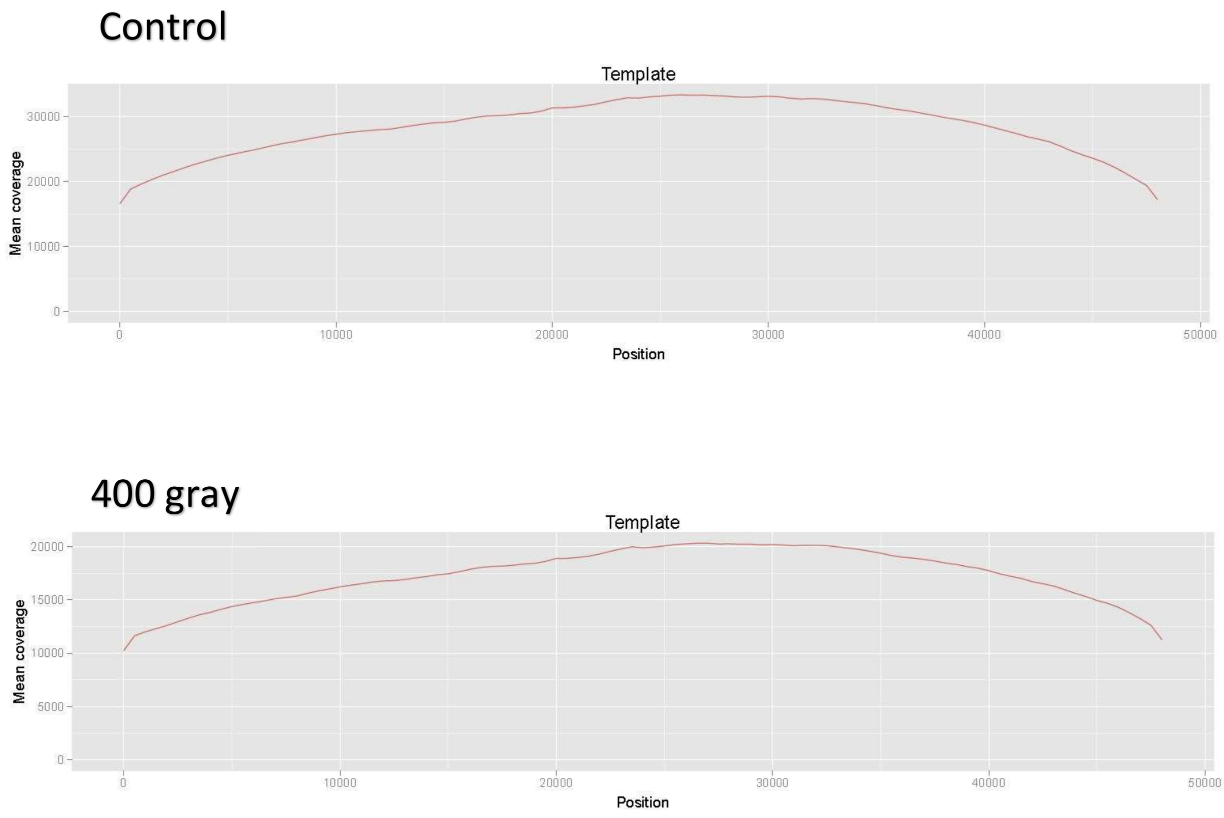

Supplementary Figure S3. Lambda genome coverage maps for FRM reagent experiments.

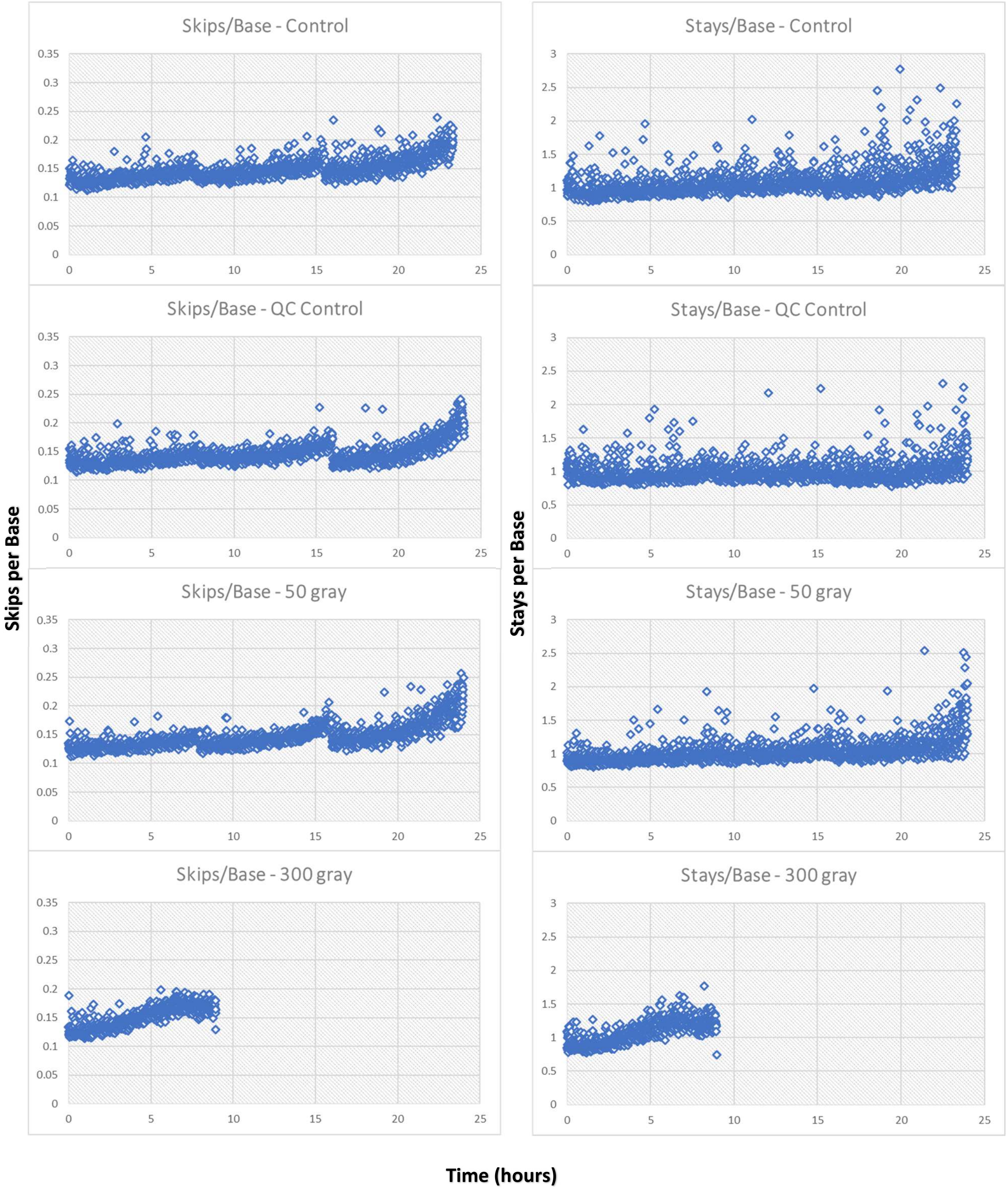

Supplementary Figure S4. Time-dependent analyses of skips, stays, read length, and electrical current detected while sequencing for flow cell experiments. Each data point represents an average value of all reads produced within a 30-second interval. Note: the vertical scale on these plots may exclude some outliers.

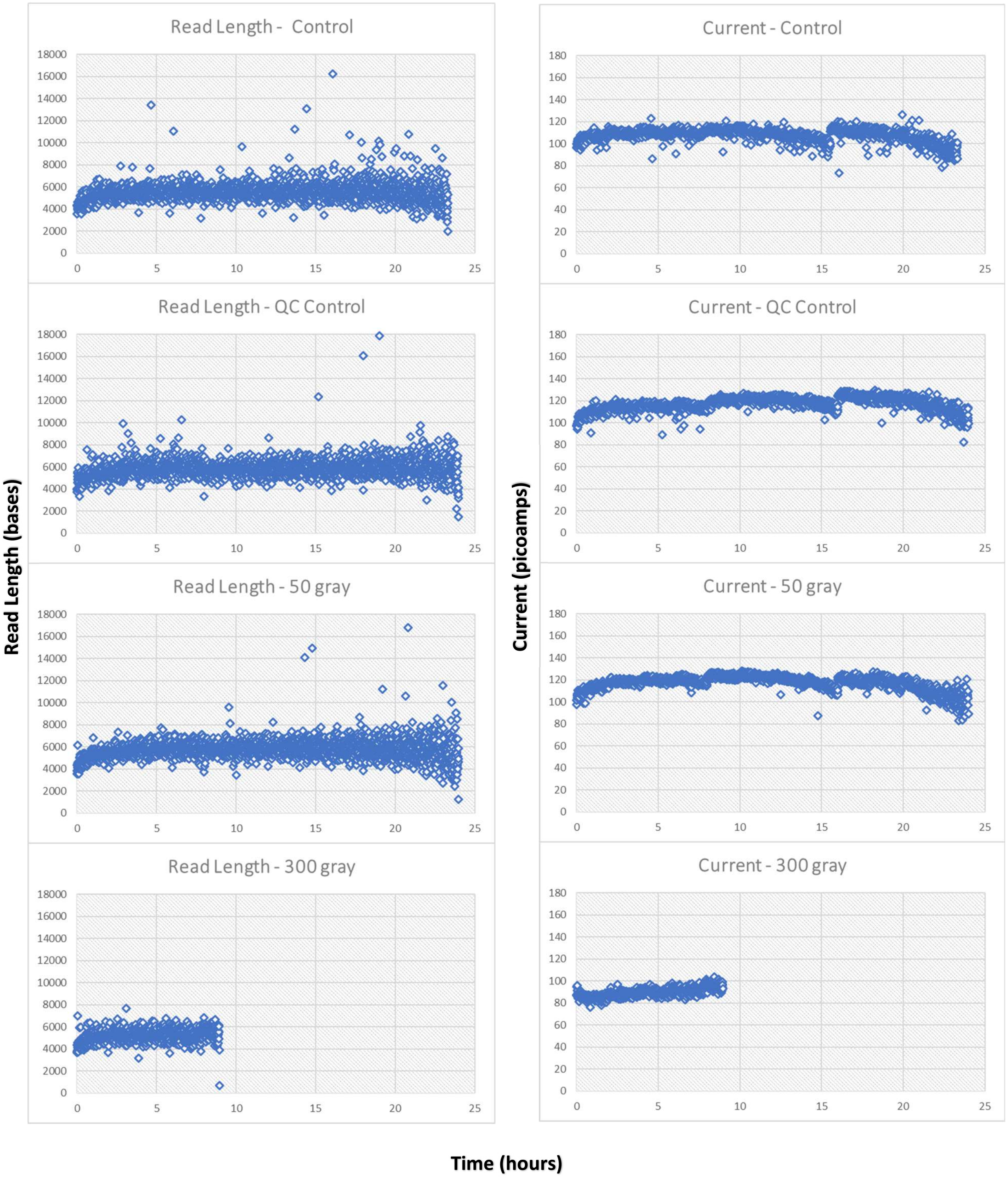

Supplementary Figure S4 (continued)

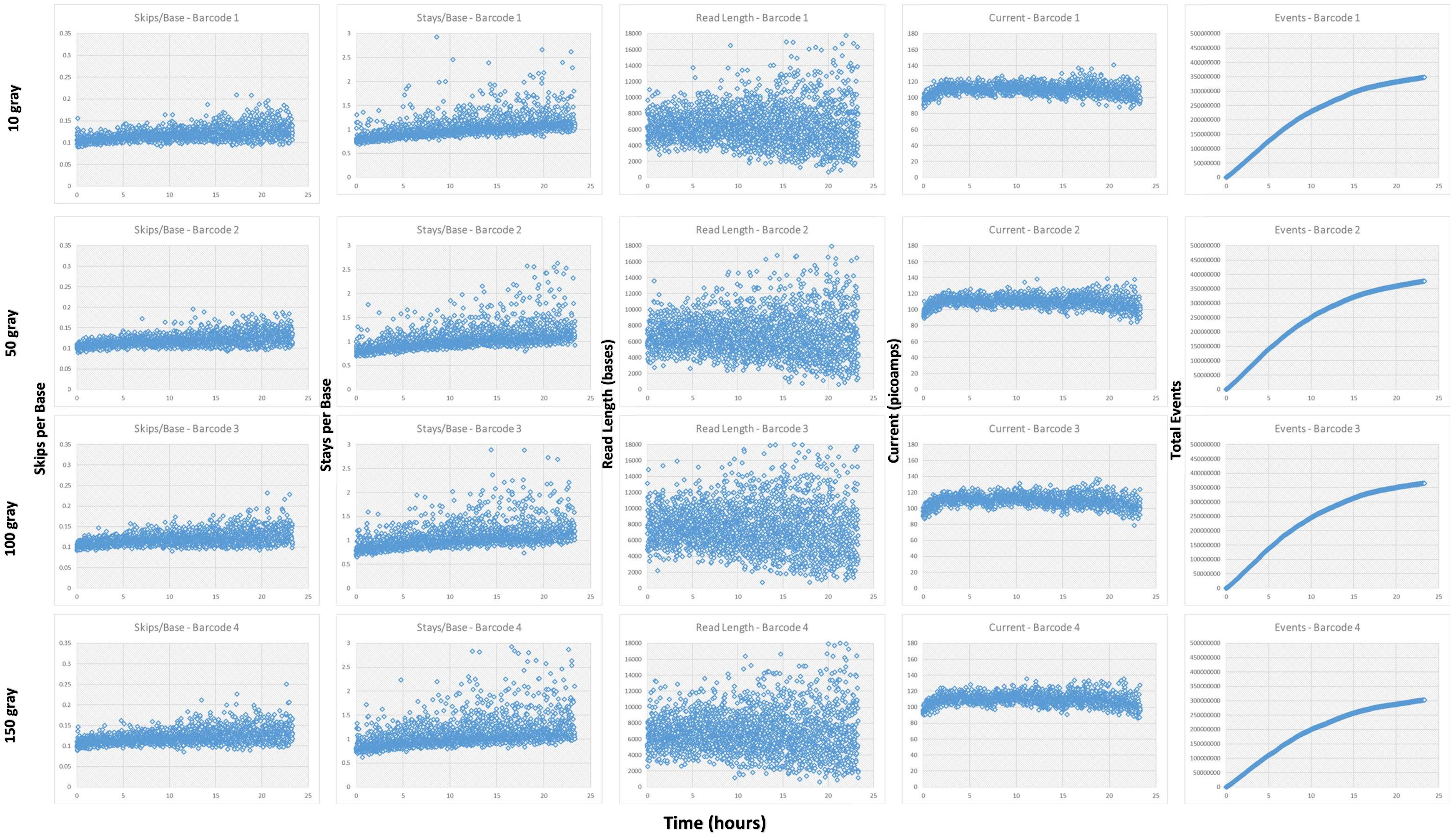

Supplementary Figure S5. Time-dependent analyses of skips, stays, read length, electrical current detected while sequencing, and cumulative total events for RAD reagent experiments. Each data point (except those on the total events plot) represents an average value of all reads produced within a 30-second interval. Note: the vertical scale on these plots may exclude some outliers.

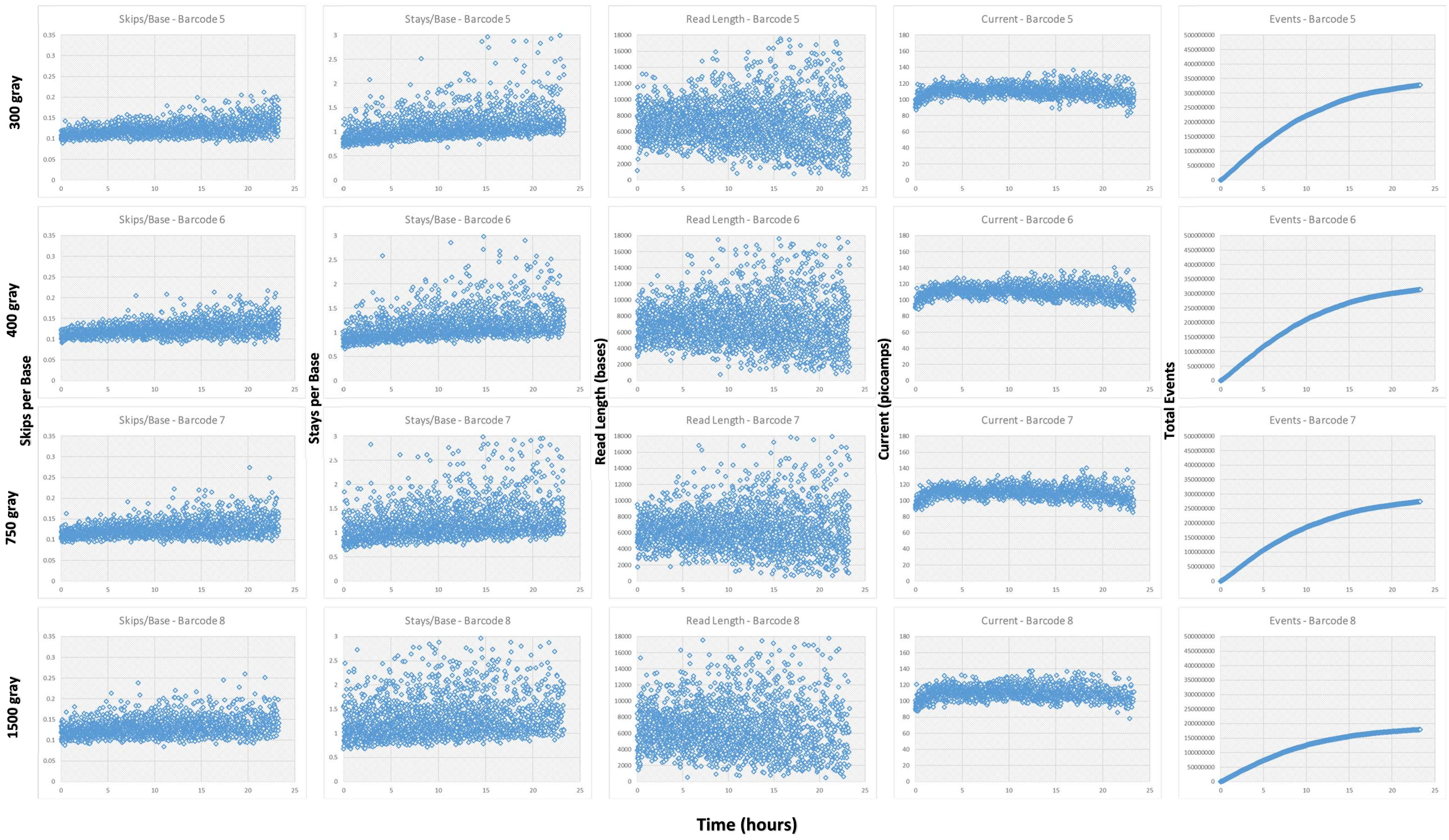

Supplementary Figure S5 (continued)

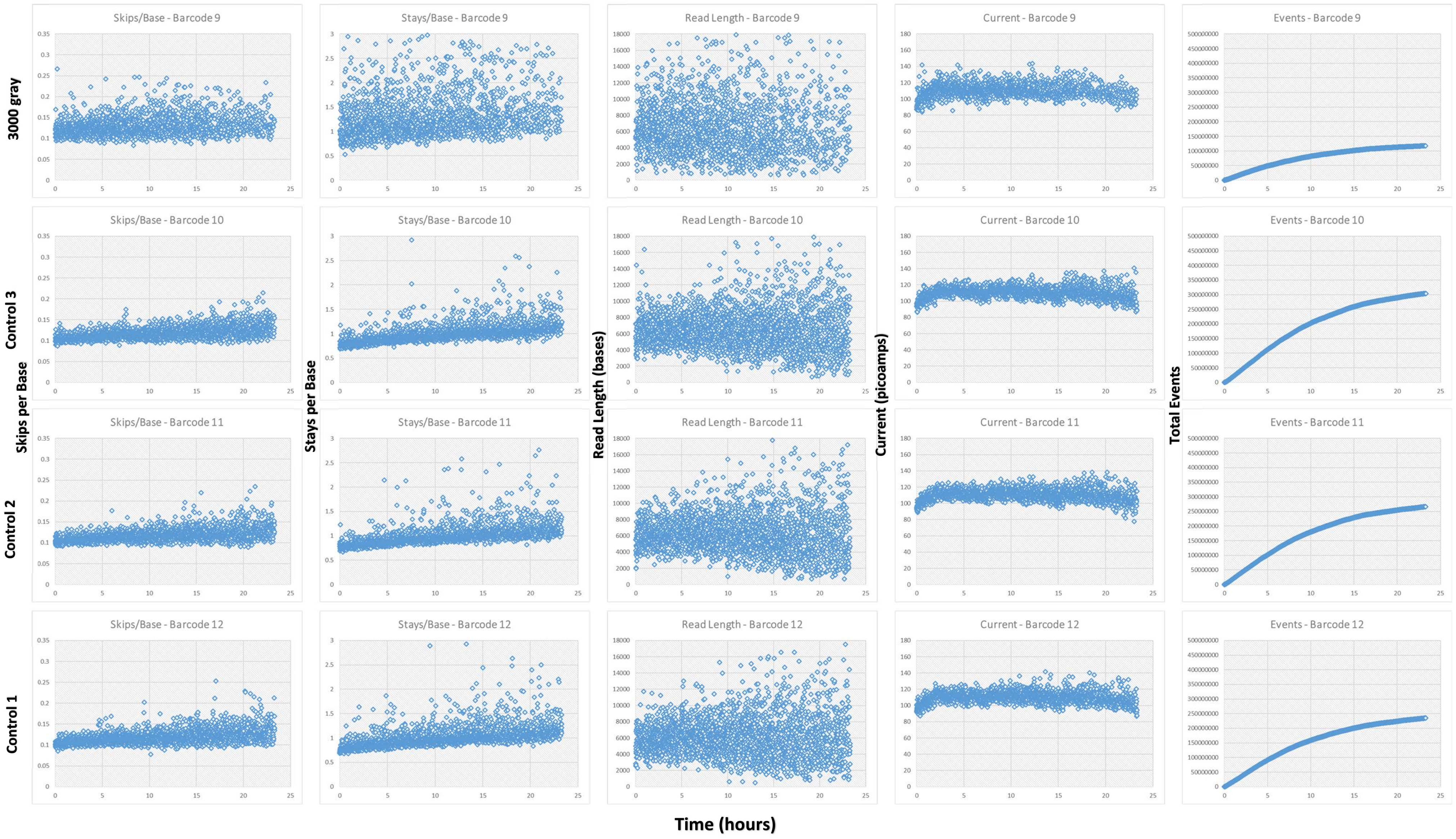

Supplementary Figure S5 (continued)

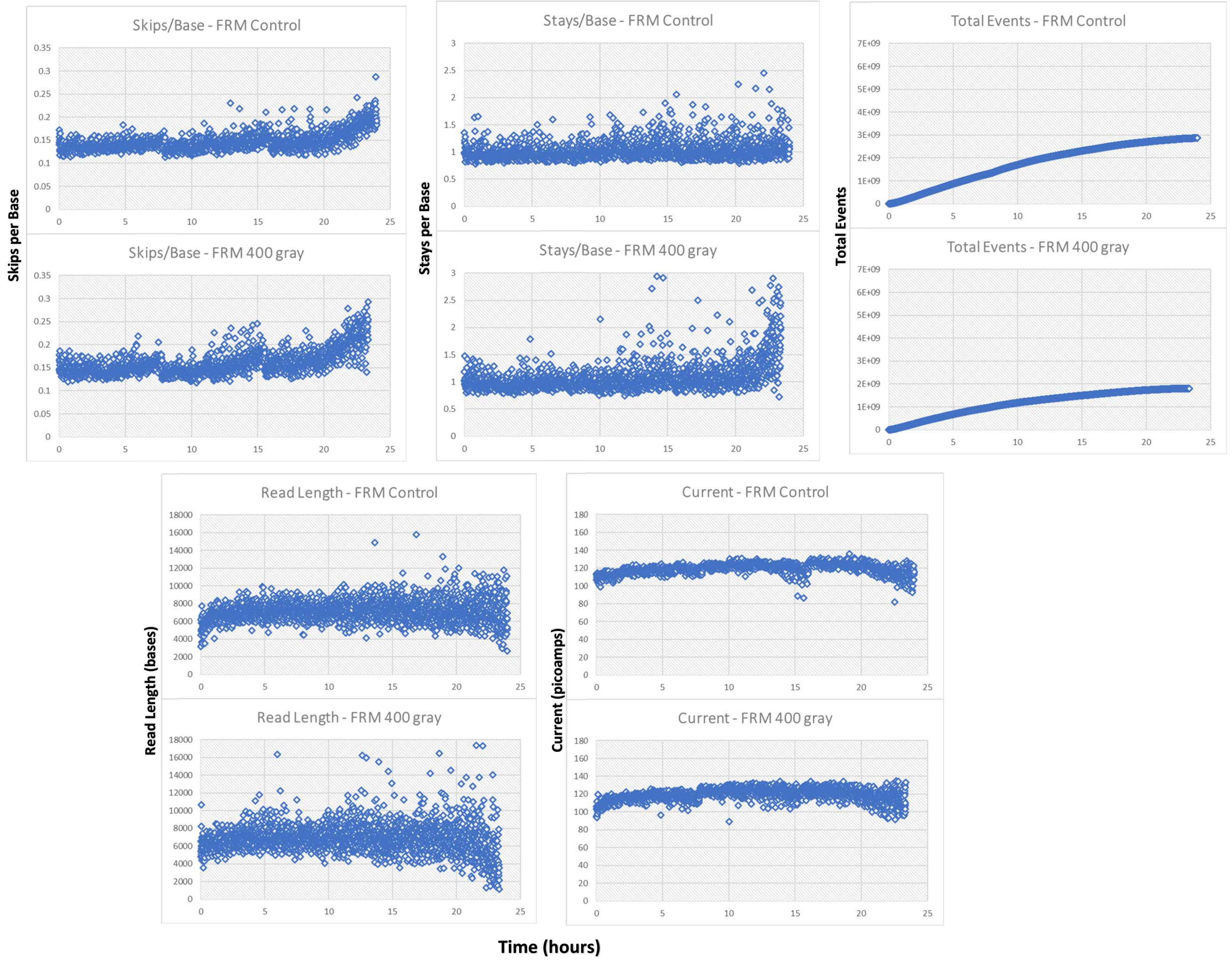

Supplementary Figure S6. Time-dependent analyses of skips, stays, cumulative total events, read length, and electrical current detected while sequencing for FRM reagent experiments. Each data point (except those on the total events plot) represents an average value of all reads produced within a 30-second interval. Note: the vertical scale on these plots may exclude some outliers.

Supplementary Table S4

RAD Reagent	Reads Produced	Reads with Alignments	Overall Base Identity	Skips per Base	Stays per Base	Average Read Length
Control	No Data					
250 gray	No Data			0.267515	6.496200	No Data
Control	161	10.56%	46.91%	0.251351	7.725801	1850.22
400 gray	203	22.17%	10.76%	0.248036	2.430298	3186.40
Control	131	6.87%	No Data	0.268698	6.416712	1625.65
600 gray	186	18.28%	15.30%	0.266575	5.677038	1853.58
Control	89	8.99%	No Data	0.229973	5.461276	1495.34
750 gray	168	21.43%	24.68%	0.283606	8.942565	1314.53
Control	94	6.38%	No Data	0.261263	6.622521	1606.88
1500 gray	86	3.49%	No Data	0.266943	10.277757	1197.88
Control	No Data					
3000 gray	No Data					

Note: skips and stays are truncated at 6 decimal places
